# Novel Bio-Based Pomelo Peel Flour/Polyethylene Glycol Composite Phase Change Material for Thermal Energy Storage

**DOI:** 10.3390/polym11122043

**Published:** 2019-12-09

**Authors:** Hai-Chen Zhang, Ben-hao Kang, Xinxin Sheng, Xiang Lu

**Affiliations:** 1 School of Materials Science & Energy Engineering, Foshan University, Foshan 528000, China; hczhang@fosu.edu.cn; 2 Key Laboratory of Polymer Processing Engineering of the Ministry of Education, National Engineering Research Center of Novel Equipment for Polymer Processing, Guangdong Key Laboratory of Technique and Equipment for Macromolecular Advanced Manufacturing, South China University of Technology, Guangzhou 510641, China; hnlgkbh@163.com; 3 Guangdong Provincial Key Laboratory of Functional Soft Condensed Matter, School of Materials and Energy, Guangdong University of Technology, Guangzhou 510006, China; 4 Department of Mechanical and Aerospace Engineering, The Hong Kong University of Science and Technology, Clear Water Bay, Hong Kong, China

**Keywords:** phase change materials, pomelo peel flour, poly (ethylene glycol), solar thermal energy storage, waste heat recovery

## Abstract

A series of novel bio-based form stable composite phase-change materials (fs-CPCMs) for solar thermal energy storage and management applications were prepared, using the pomelo peel flour (PPF) as the supporting matrix and poly (ethylene glycol) (PEG) or isocyanate-terminated PEG to induce a phase change. The microscopic structure, crystalline structures and morphologies, phase change properties, thermal stability, light-to-thermal conversion behavior, and thermal management characteristics of the obtained fs-CPCMs were studied. The results indicate that the obtained fs-CPCM-2 presented remarkable phase-change performance and high thermal stability. The melting latent heat and crystallization heat for fs-CPCM-2 are 143.2 J/g and 141.8 J/g, respectively, and its relative enthalpy efficiency (λ) is 87.4%, which are higher than most reported values in the related literature. The obtained novel bio-based fs-CPCM-2 demonstrated good potential for applications in solar thermal energy storage and waste heat recovery.

## 1. Introduction

The development of human society is closely related to the utilization of energy [1,2]. From the age of wood as the main energy source to the age of coal, and to the age of oil, every energy innovation has brought tremendous social development and new breakthroughs in science and technology [3,4]. The development and utilization of energy remarkably changed the lifestyle and living standards of human beings; however, human beings’ dependence on energy continues to increase [5]. Therefore, the energy issue has received worldwide attention. In the face of depletion of fossil fuels and the deterioration of the environment, it is extremely urgent to adjust the energy structure and find new energy sources [6,7]. The application of energy-storage technology can effectively improve the energy utilization efficiency. It is estimated that the solar radiation to the earth each year is equivalent to burning 137 trillion tons of standard coal. However, humanity can only capture and utilize a small fraction of this solar energy due to seasonality, weather changes, and other factors [8,9,10]. In addition, a large amount of energy is wasted in the form of waste heat energy in the industrial production process. Hence, due to the desire to generate and utilize more renewable energy, an important part of global sustainable development is to achieve efficient capture, conversion, and storage of solar energy at a low cost. 

In recent years, thermal energy storage (TES) technology based on phase-change materials (PCMs) has shown great potential in solar energy storage because of its high energy storage density and almost constant phase transition temperature [11,12,13,14]. Polyethylene glycol (PEG) is a typical organic PCM suitable for photo-thermal conversion and thermal energy storage due to the fact that it has a high phase change enthalpy (up to 180 J/g), an easily regulated phase change temperature, is environmentally friendly and chemically stable, and so on. [15,16,17,18]. However, PEG has good melt flow ability and all of these methods have their limitations—the risk of deformation failure and leakage during the phase change in practical applications.

To solve these problems, in recent years, a series of transfections has been used to prepare PEG-based stable composite PCMs (fs-CPCMs), which can ensure that deformation does not occur during the phase transformation. Xia et al. [13] conducted a series of novel PEG-based polyurethane (PU)/graphene oxide (GO) fs-CPCMs in which they mixed poly(ethylene glycol) (PEG) as the phase change ingredient, 4,4’-diphenylmethan diisocyanate (MDI) as the cross-linking agent, and GO as the skeleton material, which exhibited high thermal conductivity and excellent thermal stability for thermal energy storage applications. Zhou et al. [19] prepared novel PEG-based PU/halloysite nanotubes (HNTs) fs-CPCMs with excellent thermal stability, in which the HNTs not only worked as a crosslinking agent, but also a heterogeneous nucleating agent to extend the latent heat of the composite. Apparently, various novel PEG-based fs-CPCMs with high thermal performance could be obtained by chemical synthesis.

In addition, porous materials are often used as the skeleton material to improve the shape stability of PEG-based PCMs, which has attracted wide attention from researchers due to its simple preparation process via a vacuum impregnation approach. Wang et al. [20] obtained a series of PEG-based fs-CPCMs with PEG as the phase change ingredient and mesoporous silica as the supporting matrix. Likewise, Yang et al. [21] prepared PEG/expanded graphite (EG) fs-CPCMs with excellent thermal storage capacity and sealing performance, which were then used in thermal comfort improvement and indoor energy savings. Using GO and boron nitride (BN) as the supporting matrix and thermally conductive filler, Yang et al. [16] prepared a series of PEG/GO/BN fs-CPCMs which have high shape stability and high thermal conductivity by vacuum impregnation. Many similar reports have fully demonstrated that PEG-based fs-CPCMs with porous materials as supporting materials can be easily obtained by a simple approach of ordinary vacuum impregnation, which has broad potential in the industrial preparation and application of high-performance fs-CPCMs. Unfortunately, most of the porous materials used in the literature were non-biomass materials and their preparation process could damage or destroy the ecological environment more or less. Thus, it is necessary to find biomass supporting materials to prepare PEG-based fs-CPCMs with excellent phase change properties. 

Pomelo, a fruit highly prized throughout the world, is rich in water, vitamin C, and carotene. A large amount of pomelo pulp is consumed by humans every year, with most of the remaining pomelo peel subsequently thrown away as garbage, which not only increases the burden of environmental protection, but is also a great waste of resources. Actually, as a degradable biomass resource, pomelo peel can be used as an adsorption or filtration material because of its fluffy porous structure. In this study, we introduced pomelo peel flour (PPF) as supporting material and prepared a series of novel PEG-based fs-CPCMs via a vacuum impregnation approach. In order to improve the effective content of PEG in the PEG/PPF fs-CPCMs, we performed an isocyanate (–NCO) end-capping treatment on PEG, and also compared the difference in adsorption performance of PPF on PEG and NCO-terminated PEG. Meanwhile, the thermal behaviors and solar thermal storage performance of the obtained bio-based PEG/PPF fs-CPCMs were investigated. In summary, this is the first reported use of PPF as the supporting skeleton to design bio-based PEG/PPF fs-CPCMs. The results of this work will provide an explanatory theory for broadening the application of PPF in the production of high-performance bio-based fs-CPCMs, which also has important scientific and practical significance.

## 2. Materials and Methods

### 2.1. Raw Materials

Polyethylene glycol (number-average molecular weight is 6000 g/mol, PEG-6000) with a $T_{m}$ of approximately 60 °C was provided by Amresco Co. Ltd. (Radnor, PA, USA). Analytical-grade aliphatic hexamethylene diisocyanate (HDI, 99.6%) and dibutyl tin dilaurate (catalyst) were purchased from Aladdin Chemistry Co. Ltd. (Shanghai, China). Pomelo peel flour (PPF) with an average particle size of about 55 μm was provided by Xi’an Tianrui Biotechnology Co., Ltd. (Xi’an, China).

### 2.2. Preparation Methods for Fs-CPCM-1 and Fs-CPCM-2

Firstly, the isocyanate (–NCO)-terminated PEG (–NCO~PEG) was synthesized via reaction of PEG-6000 and HDI. Typically, PEG and HDI at a molar ratio of 1:2 were stirred at 80 °C for 8 h under a nitrogen atmosphere in an oil-bath with a small amount of dibutyl tin dilaurate as active catalyst.

**Scheme 1:**
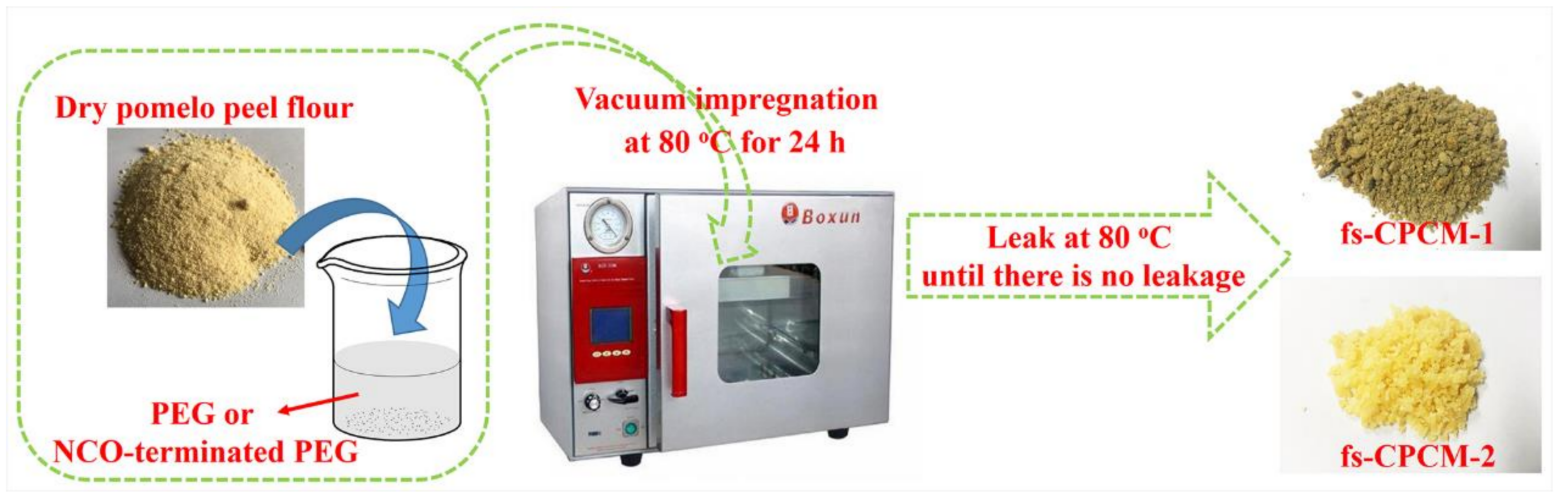
The preparation process for the bio-based form stable composite phase-change materials (fs-CPCMs).

Secondly, PEG or –NCO~PEG was blended with PPF, and, later, the mixtures were placed at 80 °C for 24 h under high vacuum to achieve the adsorption saturation. Subsequently, the specimens were wrapped by a piece of filter paper and heated at 80 °C for 2 h to remove excess PEG or –NCO~PEG. The filter paper was replaced regularly until no PEG or –NCO~PEG leakage from the specimen occurred. The preparation process is shown in Scheme 1.

Finally, PEG/PPF and -NCO~PEG/PPF fs-CPCMs were obtained and identified as fs-CPCM-1 and fs-CPCM-2, respectively. The effective content of PEG segment in fs-CPCM-1 ($w_{1}$) and fs-CPCM-2 ($w_{2}$) were calculated by Equations (1) and (2), respectively:

(1) $$w_{1}=\frac{m_{12}-m_{11}}{m_{12}}\times 100\%,$$

(2) $$w_{2}=\frac{m_{22}-m_{21}}{m_{22}}\times 94.7\%\times 100\%,$$

where $m_{11}$ and $m_{21}$ are the initial PPF mass in fs-CPCM-1 and fs-CPCM-2, respectively, and $m_{12}$ and $m_{22}$ are the mass of the final fs-CPCM-1 and fs-CPCM-2 product, respectively. In addition, for fs-CPCM-2, the 94.7% (calculated according to the molar ratio of PEG to HDI) is the PEG segment content in the obtained –NCO~PEG. The calculation results show that the maximum adsorption mass fraction of PEG segment in fs-CPCM-1 and fs-CPCM-2 were 49.2 and 89.9 wt %, respectively.

### 2.3. Analysis Methods

The morphologies of PPF, fs-CPCM-1, and fs-CPCM-2 were observed by a scanning electron microscope (SEM, Quanta, FEG 250) with an accelerating voltage of 5 kV. All the samples were gold-sputtered before the observation.

The specific surface area and pore volume of PPF were determined at 77 K using a BELSORP-max equipment (Microtrac BEL, Osaka, Japan) based on the Brunauer–Emmett–Teller (BET) method.

The FT-IR spectra of the samples were recorded using a Spectrum 2000 instrument (USA) with a resolution of 2 cm^−1^ and 32 scans in the wavenumber range of 4000–400 cm^−1^. Before testing, pure PEG, PPF, and two types of fs-CPCMs samples were mixed separately with KBr powder and cold-pressed into thin films for measurement. 

The crystal structure of the pure PEG and obtained fs-CPCMs were examined via a wide-angle X-ray diffractometer (WAXD, Bruker D8 Advance, Karlsruhe, Germany) from 2θ of 5° to 50° at a scanning rate of 2°/min.

The crystal morphology of the pure PEG and obtained fs-CPCMs were observed using a polarized optical microscope (POM, Axioskop 40POL, Germany) equipped with a high-resolution CCD camera.

The phase change properties, including the phase change temperatures and phase change enthalpies, of all samples were determined by a differential scanning calorimeter (DSC, NETZSCH DSC 204, Germany) from 0 to 100 °C at a heating/cooling rate of 10 °C/min^−1^ under a nitrogen atmosphere.

The thermal reliability and reusability of the obtained fs-CPCMs were studied by accelerated thermal cycling testing via a high–low temperature chamber (KSON KTHB-415TBS, Taiwan, China) in accordance with the method described in our previous work [22]. Afterwards, DSC were used for examining the phase change properties of the obtained fs-CPCMs after accelerated thermal cycles.

The thermal stability of pure PEG, PPF, fs-CPCM-1 and fs-CPCM-2 were investigated by thermogravimetric analysis (TGA, NETZSCH TG209) in the temperature range of 30–700 °C at a heating rate of 10 °C /min under a nitrogen atmosphere. All of the thermal stability parameters were acquired in five repeats.

The solar thermal energy storage behavior of the obtained fs-CPCM-1 and fs-CPCM-2 were studied via the self-built light–thermal conversion experimental device, including a 500 W infrared light (used to simulated the solar irradiation) and an infrared thermography camera (FLIR, SC 3000, City, US State abbrev. if applicable, Country, used to recording the temperature change of sample).

## 3. Results and Discussion

### 3.1. Morphology and Structure of PPF, Fs-CPCM-1, and Fs-CPCM-2

**Figure 1:**
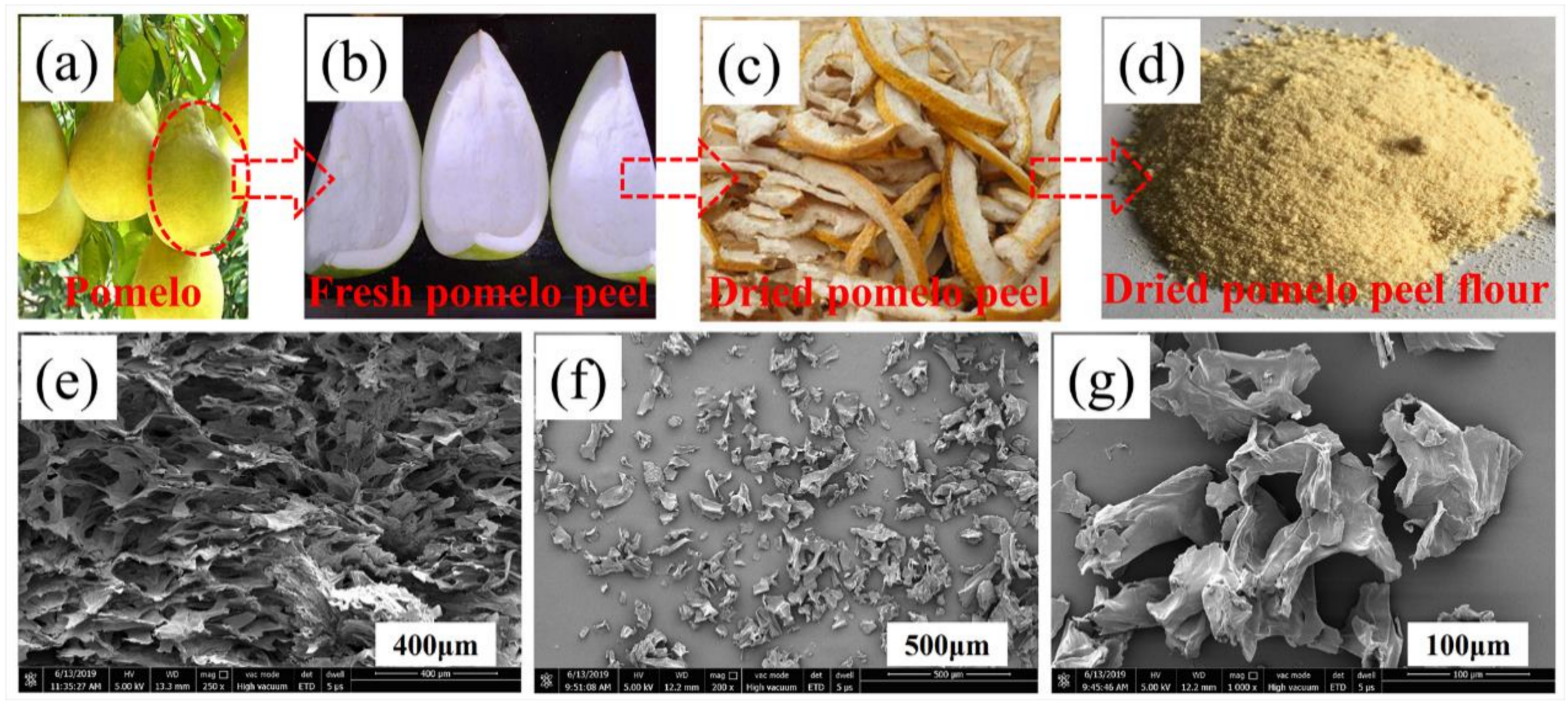
The digital photo of (**a**) pomelo, (**b**,**c**) pomelo peel, and (**d**) pomelo peel flour, and SEM images of (**e**) pomelo peel and (**f**,**g**) pomelo peel flour.

Figure 1a,b present the digital pictures of fresh pomelo and pomelo peel, respectively. In order to use pomelo peel as a supporting material for PCMs more efficiently and conveniently, it is necessary to remove the moisture contained in it and break it into pomelo peel flour (PPF) before use. Figure 1c,e show the digital picture and SEM images of dried pomelo peel, respectively. Figure 1d,f,g show the digital photo and SEM image of dried PPF, respectively. It can be clearly seen that the pomelo peel and PPF are typical fluffy porous materials, which can be used as supporting materials for PCMs. In addition, due to the high water content in the pomelo peel, there must be a large amount of hydrophilic groups (hydroxy or carboxyl) on the surface of the PPF. Due to the uneven surface, hydrophilicity and porosity of PPF, it can be joined to hydrophilic PEG or –NCO~PEG through hydrogen bonding, capillary force, surface tension force, and chemical covalent bonds.

**Figure 2:**
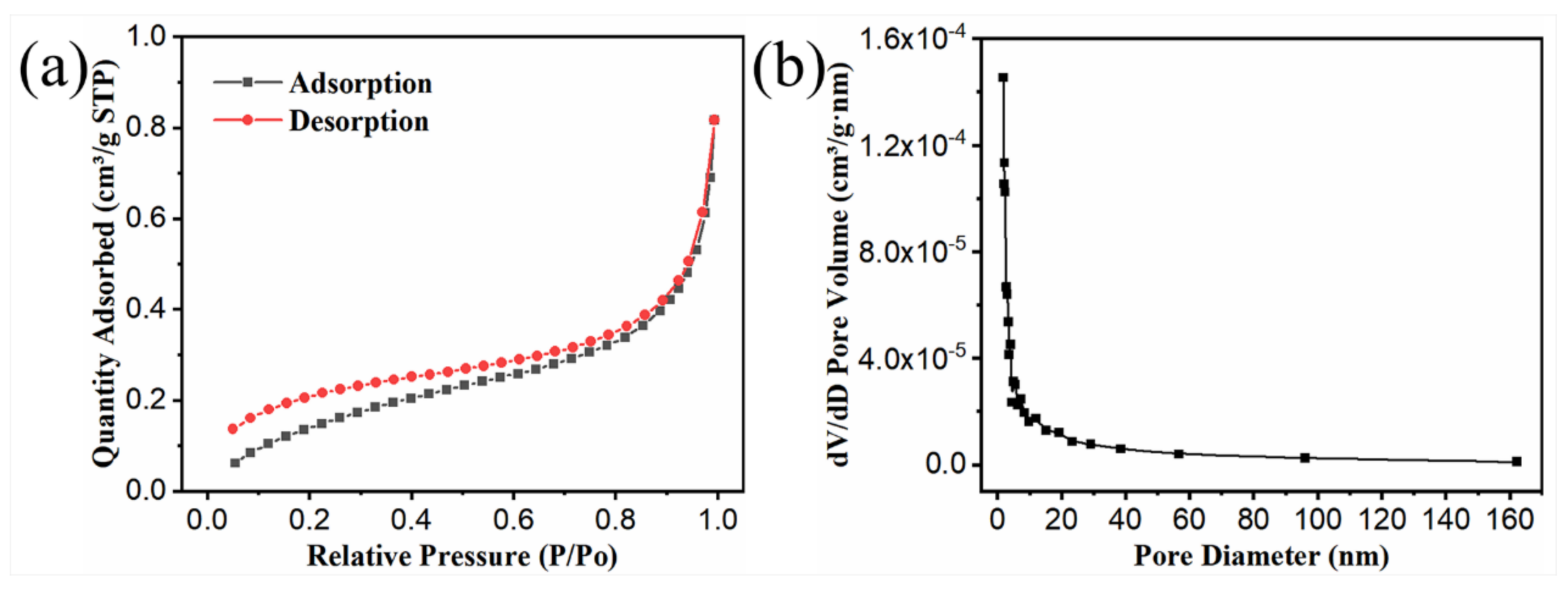
(**a**) the nitrogen adsorption/desorption curves and (**b**) pore diameter distribution of pomelo peel flour (PPF).

The Brunauer–Emmett–Teller (BET) method is widely applied to characterize the adsorption properties of the particle surface. Figure 2a,b are the nitrogen adsorption/desorption curves and pore diameter distribution of PPF, respectively. It shows that the obtained PPF has a wide range of pore diameter (1 to 160 nm), and the calculated average pore diameter and specific surface area for PPF are about 7.1 nm and 0.65 m^2^/g. These results indicate that PPF is a typical mesoporous material, and these mesoporous structures facilitate the absorption of molted PEG fragments by PPF [23]. 

**Figure 3:**
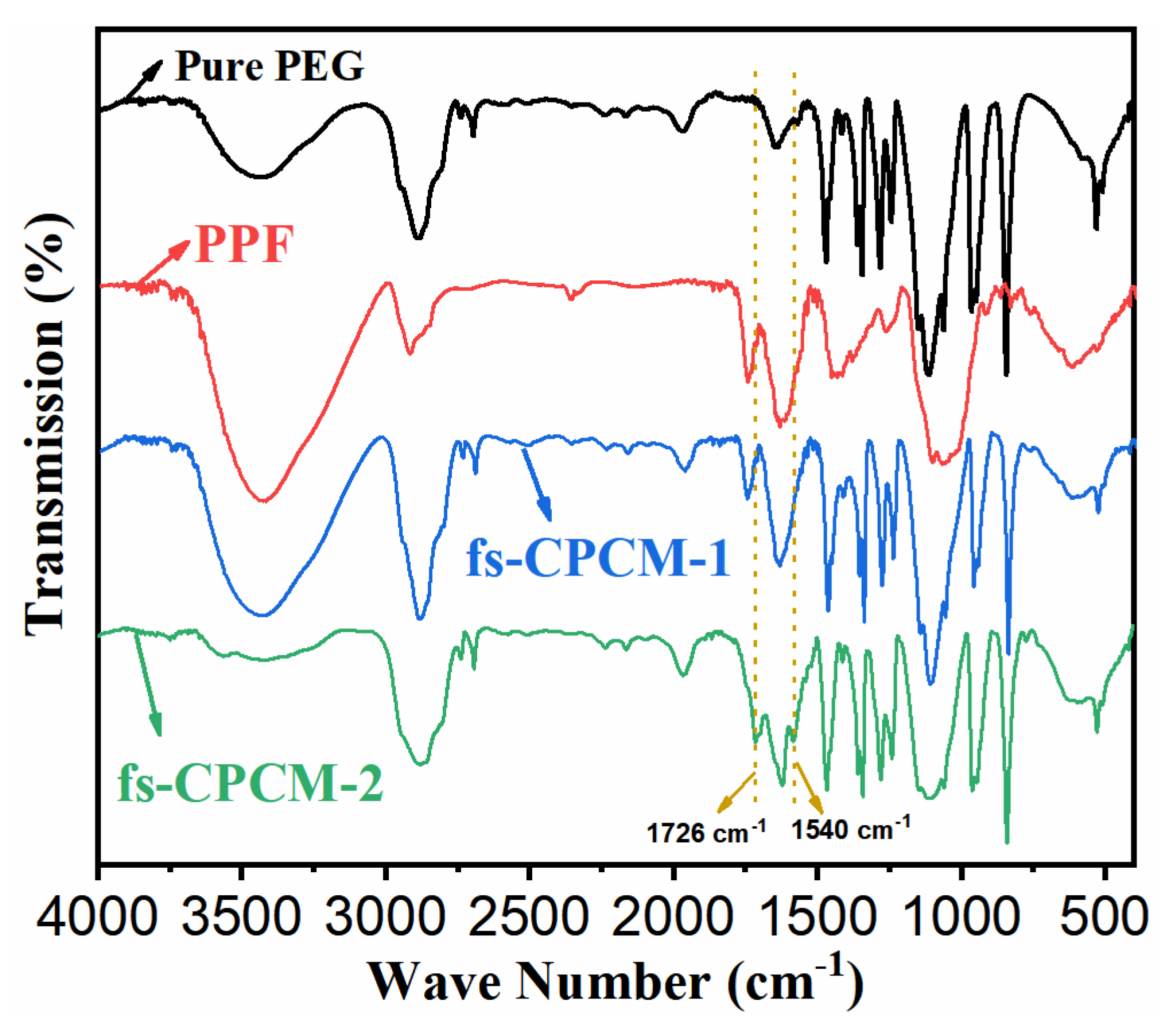
The FT-IR spectra of pure poly (ethylene glycol) (PEG), pomelo peel flour (PPF), fs-CPCM-1 and fs-CPCM-2.

Figure 3 provides the FT-IR spectra of pure PEG, PPF, fs-CPCM-1, and fs-CPCM-2. In the FT-IR spectra of pure PEG, the stretching vibration characteristic absorption peaks for –O–H and –C–O appear at 3440 and 1107 cm^−1^, respectively. The absorption peaks for C–H bonds appear at 2887, 1465, 1355, 1277, 964, and 846 cm^−1^ [24,25]. For PPF, the characteristic peaks at 3438 and 1108 cm^−1^ correspond to the stretching vibration of –O–H and –C–O, respectively, indicating the presence of a large number of hydrophilic groups on the surface of the PPF [23]. In contrast, the FT-IR spectrum of fs-CPCM-1 is a simple superposition of the PEG and PPF spectra, and no significant new characteristic peaks are presented. This indicates that there are no chemical covalent bonds between PEG and PPF in the fs-CPCM-1, but a simple physical absorption. Compared with fs-CPCM-1, there are two new peaks that appear at around 1540 and 1726 cm^−1^ for fs-CPCM-2, which correspond to the amide and carbonyl vibration of –NHCOO– groups. Meanwhile, the characteristic peak of active –NCO from –NCO~PEG at around 2265 cm^−1^ disappears entirely and the characteristic peaks for –OH of PEG and PPF become weak. The FT-IR results indicate that the –NCO~PEG has successfully reacted with the PPF, which is consistent with the reports in [22]. 

**Figure 4:**
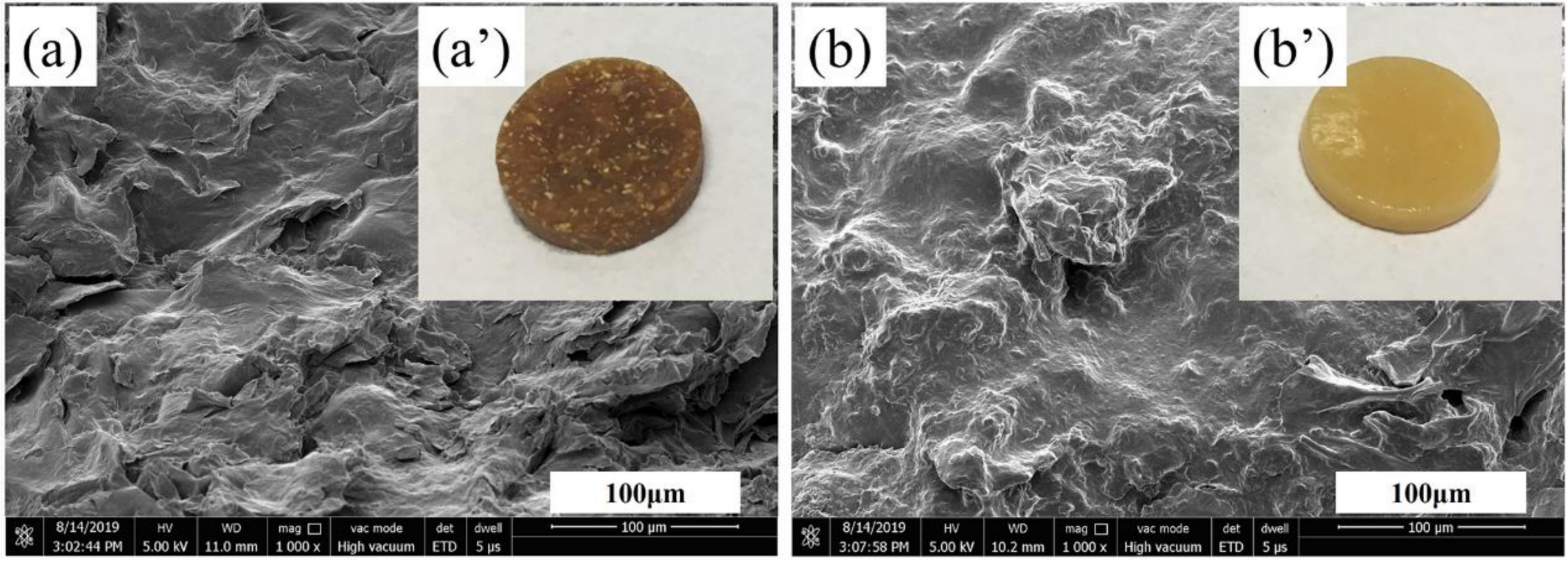
The SEM images of (**a**) fs-CPCM-1 and (**b**) fs-CPCM-2, and digital photo of (**a’**) fs-CPCM-1 and (**b’**) fs-CPCM-2 at 80 °C for 1 h.

Supported by the calculation results, the maximum adsorption mass fraction of PEG segment in the fs-CPCM-1 and fs-CPCM-2 samples were 49.2 and 89.9 wt %, respectively. The effective content of PEG segment in fs-CPCM-2 is higher than that in fs-CPCM-1, which could be attributed to the stronger interface interaction between –NCO~PEG and PPF. Compared to fs-CPCM-1, in addition to the hydrogen bonding, surface tension force, and capillary force, there are also chemical interface interactions between PPF and the PEG segment via –NHCOO– groups in fs-CPCM-2. Thus, there is an evident phase interface between PEG and PPF in fs-CPCM-1 (Figure 4a), but a blurred phase interface between PEG and PPF in fs-CPCM-2 due to the stronger interface interaction (Figure 4b). In addition, as shown in Figure 4a’,b’, there is no leakage in fs-CPCM-1 and fs-CPCM-2 after heat treatment at 80 °C for 1 h. 

### 3.2. Crystalline Performances

**Figure 5:**
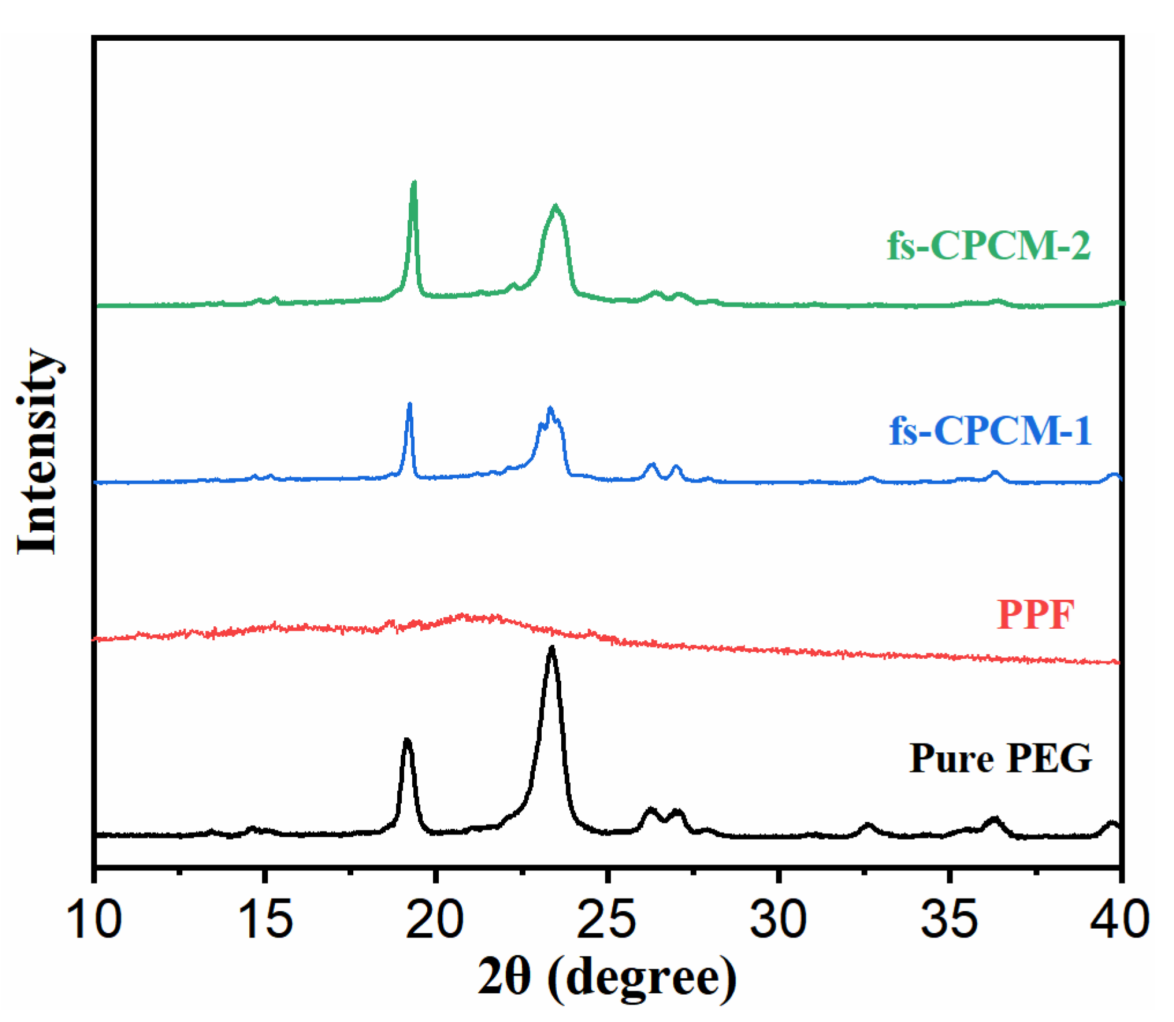
The WAXD patterns of pure PEG, PPF, fs-CPCM-1 and fs-CPCM-2.

**Figure 6:**
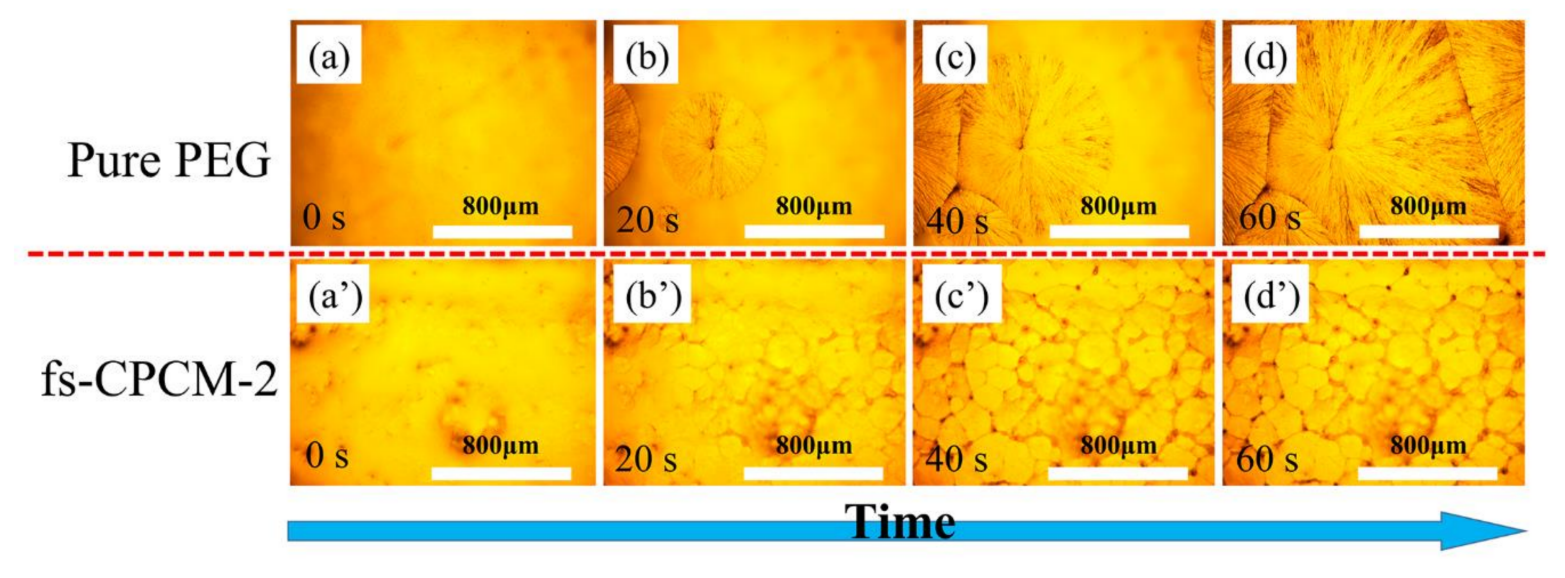
POM images of (**a**–**d**) pure PEG and (**a’**–**d’**) fs-CPCM-2 cooled from 100 °C to room temperature by natural cooling.

WAXD and POM were used to verify the crystal structure and crystal morphology of the bio-based PEG/PPF CPCMs. Figure 5 shows the WAXD patterns of pure PEG, PPF, fs-CPCM-1, and fs-CPCM-2. For pure PEG, the two intense and sharp peaks at about 19.1 and 23.5 degrees two-theta belong to the diffractions of (120) and (132) crystal planes, and their intensity and sharpness indicate the superior crystalline structure of pure PEG [19]. However, only one broad band is observed from 19.0 to 25.0 degrees two-theta for PPF, indicating poor crystallization of this sample. For fs-CPCM-1 and fs-CPCM-2, both their WAXD patterns contain all the characteristic diffraction peaks of pure PEG with the absence of any new peaks. However, the peak intensity of fs-CPCM-1 and fs-CPCM-2 at 19.1 and 23.5 degrees two-theta are lower than that of pure PEG, which implies the destruction of the crystal structure of PEG segments due to the introduction of PPF in fs-CPCM-1 and fs-CPCM-2. The reason may be that the crystalline regions of PEG segments in the obtained fs-CPCM-1 and fs-CPCM-2 were restricted due to the existence of PPF (for fs-CPCM-1 and fs-CPCM-2) and formation of the crosslinking network structure (for fs-CPCM-2) [26,27]. Therefore, the orientation and arrangement of PEG segments in fs-CPCM-1 and fs-CPCM-2 are partially confined, and it is difficult for fs-CPCM-1 and fs-CPCM-2 to form perfect crystals. The POM images of the crystal growth process for pure PEG and fs-CPCM-2 during natural cooling from 100 °C to room temperature are shown in Figure 6a–d and a’–d’, respectively. Regretfully, due to the high PPF content in fs-CPCM-1 (as high as 50.8 wt %), it is difficult to prepare the test sample for POM, and no POM images of fs-CPCM-1 can be observed. For pure PEG, in the field of view, only about four, large, cross petal-shaped crystal spherulites can be observed because of its high symmetry, and its average spherulite radius is about 800 μm. However, in the same field of view, there are lots of small cross petal-shaped crystal spherulites observed for fs-CPCM-2, and its average spherulite radius is only about 100 μm. As we all know, the crystallization process of polymer can be divided into nucleation and crystal growth. In fs-CPCM-2, the existing PPF can act as a heterogeneous nucleating agent for melted PEG segments. Thus, compared with pure PEG, in the initial stage of the crystallization process, there are a large number of crystal nuclei formed for fs-CPCM-2 (as shown in Figure 6b,b’). 

### 3.3. Phase Change Properties

**Figure 7:**
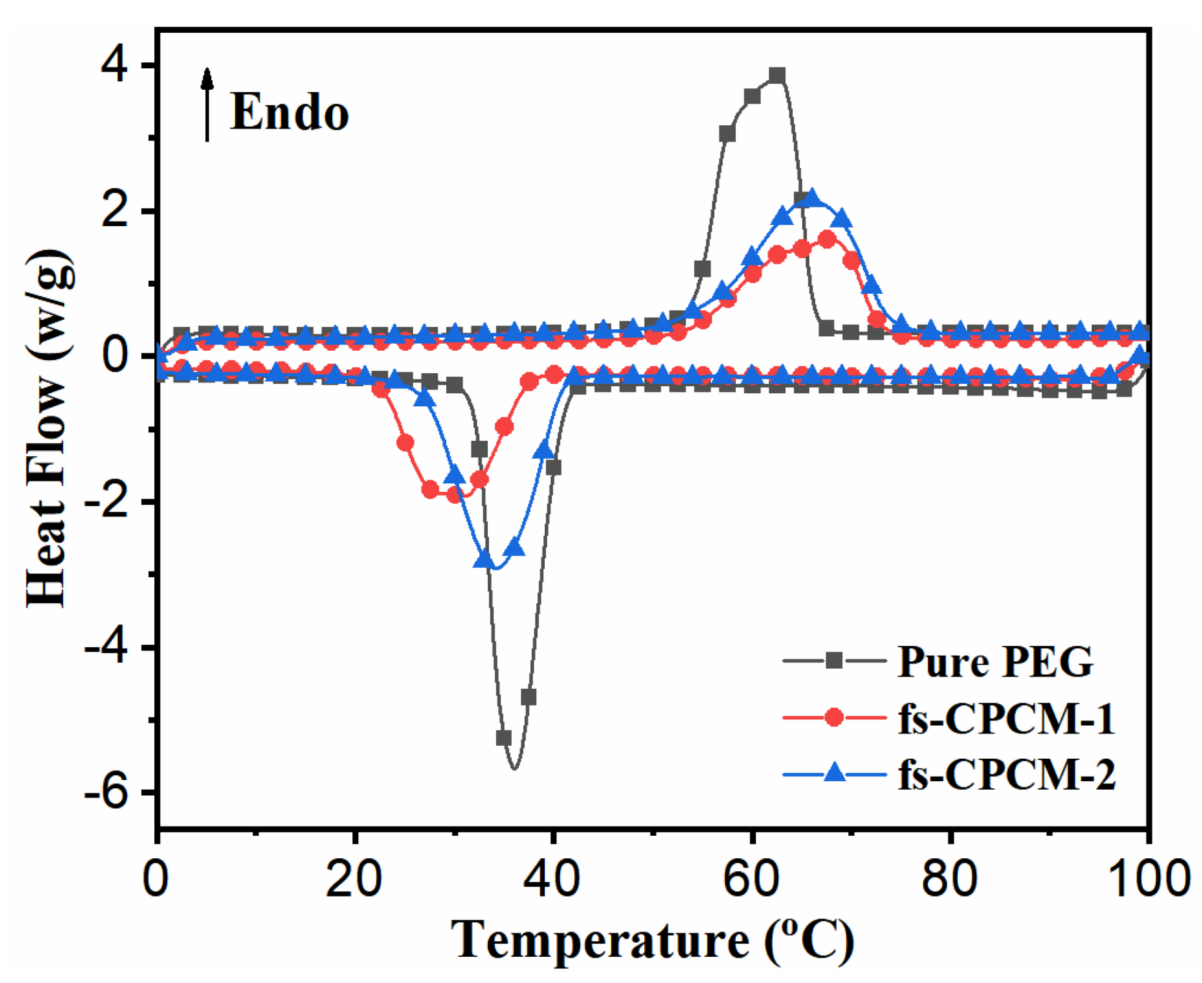
DSC curves of pure PEG and obtained fs-CPCM-1 and fs-CPCM-2.

For PCMs, the phase change properties are the most important core factors. Thus, the DSC technique is used to characterize the phase change properties of pure PEG, fs-CPCM-1, and fs-CPCM-2. The DSC curves and corresponding phase change parameters are shown and summarized in Figure 7 and Table 1. Pure PEG, fs-CPCM-1, and fs-CPCM-2 all exhibit similar melting and crystallization behaviors between 20 °C and 80 °C. Specifically, as compared with pure PEG, the melting and crystallization temperatures of the PEG segments in fs-CPCM-1 and fs-CPCM-2 changed significantly upon the introduction of PPF. Table 1 summarizes the results obtained for the onset melting temperature ($T_{mo}$), peak melting temperature ($T_{mp}$), end melting temperature ($T_{me}$), onset crystallization temperature ($T_{co}$), peak crystallization temperature ($T_{cp}$), and end crystallization temperature ($T_{ce}$) of the pure PEG, fs-CPCM-1, and fs-CPCM-2. The reason for the variation in these phase change temperatures can be attributed to the restricted crystallization of PEG segments in the obtained fs-CPCM-1 and fs-CPCM-2. In addition, due to the introduction of PPF, the melting latent heat ($∆H_{m}$) and crystallization latent heat ($∆H_{c}$) of fs-CPCM-1 are significantly reduced to 88.9 J/g and 85.1 J/g, respectively. In addition, the $∆H_{m}$ and $∆H_{c}$ of fs-CPCM-2 are reduced to 143.2 J/g and 141.8 J/g, respectively. In order to evaluate the influence of supporting materials (such as PPF) on the $∆H_{m}$ and $∆H_{c}$, the relative enthalpy efficiency (λ: the free movement degree of PEG segments) is introduced and calculated by Equation (3):

(3) $$\lambda =\frac{∆H_{m-PCM}}{∆H_{m-PEG}\times W_{f}}\times 100,$$

where $∆H_{m-PCM}$ reflects the $∆H_{m}$ of fs-CPCM-1 or fs-CPCM-2, $∆H_{m-PEG}$ reflects the $∆H_{m}$ of pure PEG, and $W_{f}$ reflects the mass fraction of PEG segments in the fs-CPCMs. For fs-CPCMs, the larger the λ value is, the smaller the latent heat loss. As exhibited in Table 2, the λ values of fs-CPCM-1 and fs-CPCM-2 are 99.1% and 87.4%, respectively, thus being clearly lower in the case of fs-CPCM-1. The reason for this is that, in addition to the physical restriction of PPF on the crystallization of PEG segments, there are also chemical restrictions between PEG segments and PPF for fs-CPCM-2, which further hindered the orientation and arrangement of PEG segments [19,28]. These DSC results are consistent with the XRD and POM results. However, the λ value and actual latent heat of fs-CPCM-2 in this work are superior to most of the traditional PEG-based fs-CPCMs that are reported in the literature (as listed in Table 2). It demonstrated that the introduction of PPF as the supporting skeleton and –NCO-terminated PEG as an ingredient is an applicable method used in fabricating novel PEG-based fs-CPCMs with comparatively high latent heat.

### 3.4. Thermal Reliability and Reusability

**Figure 8:**
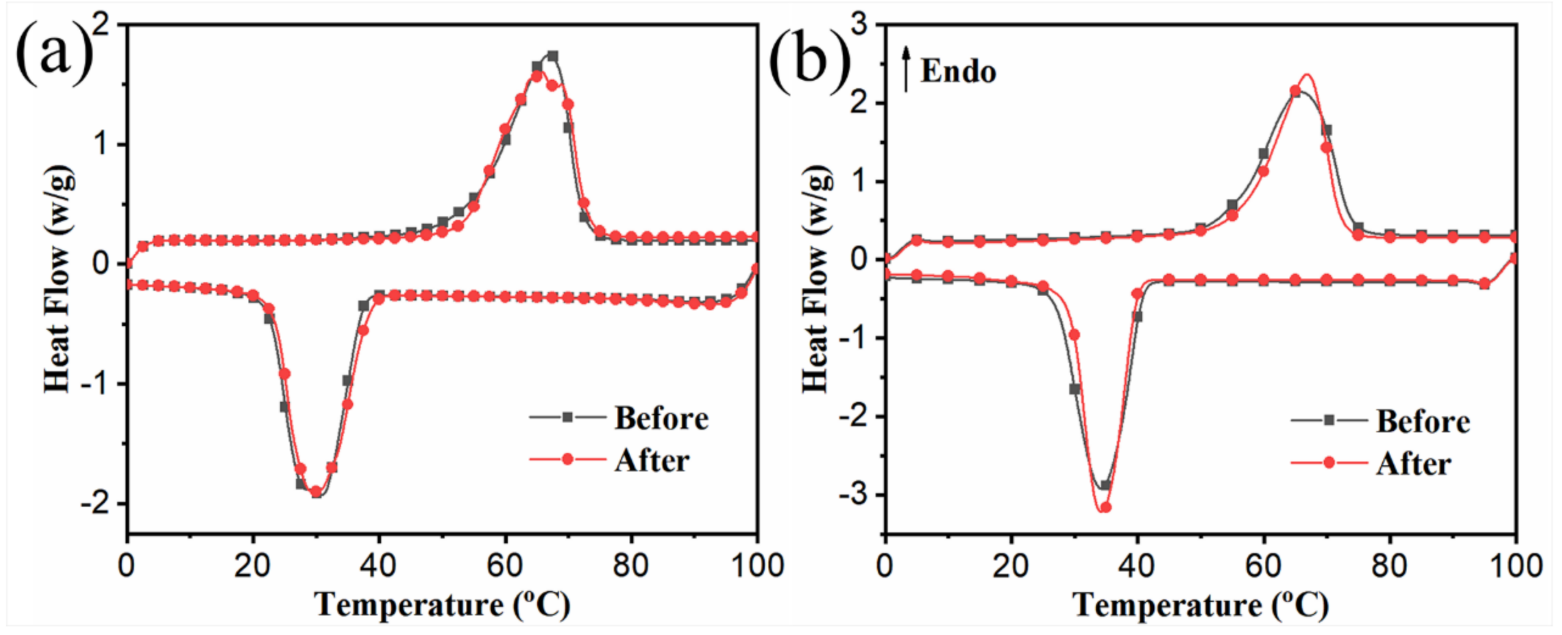
DSC curves of (**a**) fs-CPCM-1 and (**b**) fs-CPCM-2 before and after 100 thermal cycles.

In actual situations, fs-CPCMs are used multiple times. Thus, after long-term practical use, the phase change behavior of fs-CPCM-1 and fs-CPCM-2 should remain stable. In this study, accelerated thermal cycling testing was used to assess the thermal reliability and reusability of fs-CPCM-1 and fs-CPCM-2 via a high–low temperature chamber. Figure 8 and Table 3 show the DSC curves and phase change parameters of fs-CPCM-1 and fs-CPCM-2 before and after 100 thermal cycles, respectively. To evaluate the different phase change of the melting process and cooling process before and after thermal cycles, the coefficient $\eta_{m}$ (melting process) and $\eta_{c}$ (cooling process) were introduced, and calculated using Equations (4) and (5), respectively:

(4) $$\eta_{m}=\left|\frac{∆H_{mb}-∆H_{ma}}{∆H_{mb}}\right|\times 100\%,$$

(5) $$\eta_{c}=\left|\frac{∆H_{cb}-∆H_{ca}}{∆H_{cb}}\right|\times 100\%,$$

where $∆H_{mb}$ and $∆H_{ma}$ are the melting latent heat before and after thermal cycling, respectively, $∆H_{cb}$ and $∆H_{ca}$ are the crystallization latent heat before and after thermal cycling. The smaller the value of $\eta_{m}$ and $\eta_{c}$, the better the thermal reliability and reusability. From Figure 8 and Table 3, the $\eta_{m}$ and $\eta_{c}$ values for fs-CPCM-1 and fs-CPCM-2 are as low as 1.5%, 0.5% and 0.3%, 1.3%, respectively. The low $\eta_{m}$ and $\eta_{c}$ values indicate that there is no obvious change in the phase change properties for fs-CPCM-1 and fs-CPCM-2 after 100 thermal cycles, and the minor changes can be ignored for thermal energy storage applications. The excellent thermal reliability and reusability show that the obtained fs-CPCM-1 and fs-CPCM-2 are suitable for long-term practical use.

### 3.5. Light-to-Thermal Conversion and Storage

**Figure 9:**
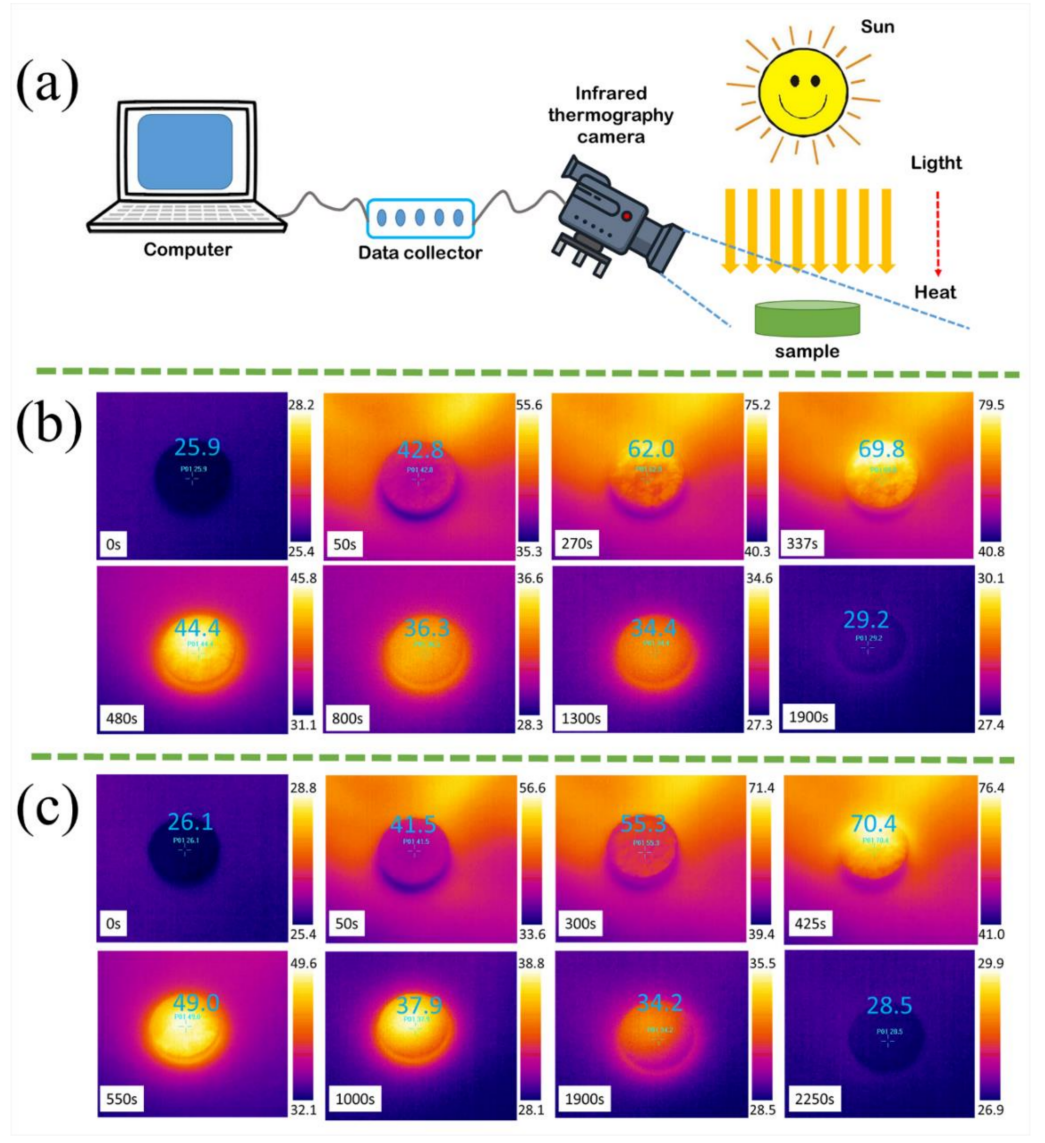
(**a**) light-to-thermal conversion and storage schematic, thermal images of (**b**) fs-CPCM-1 and (**c**) fs-CPCM-2 during the heating and cooling process.

**Figure 10:**
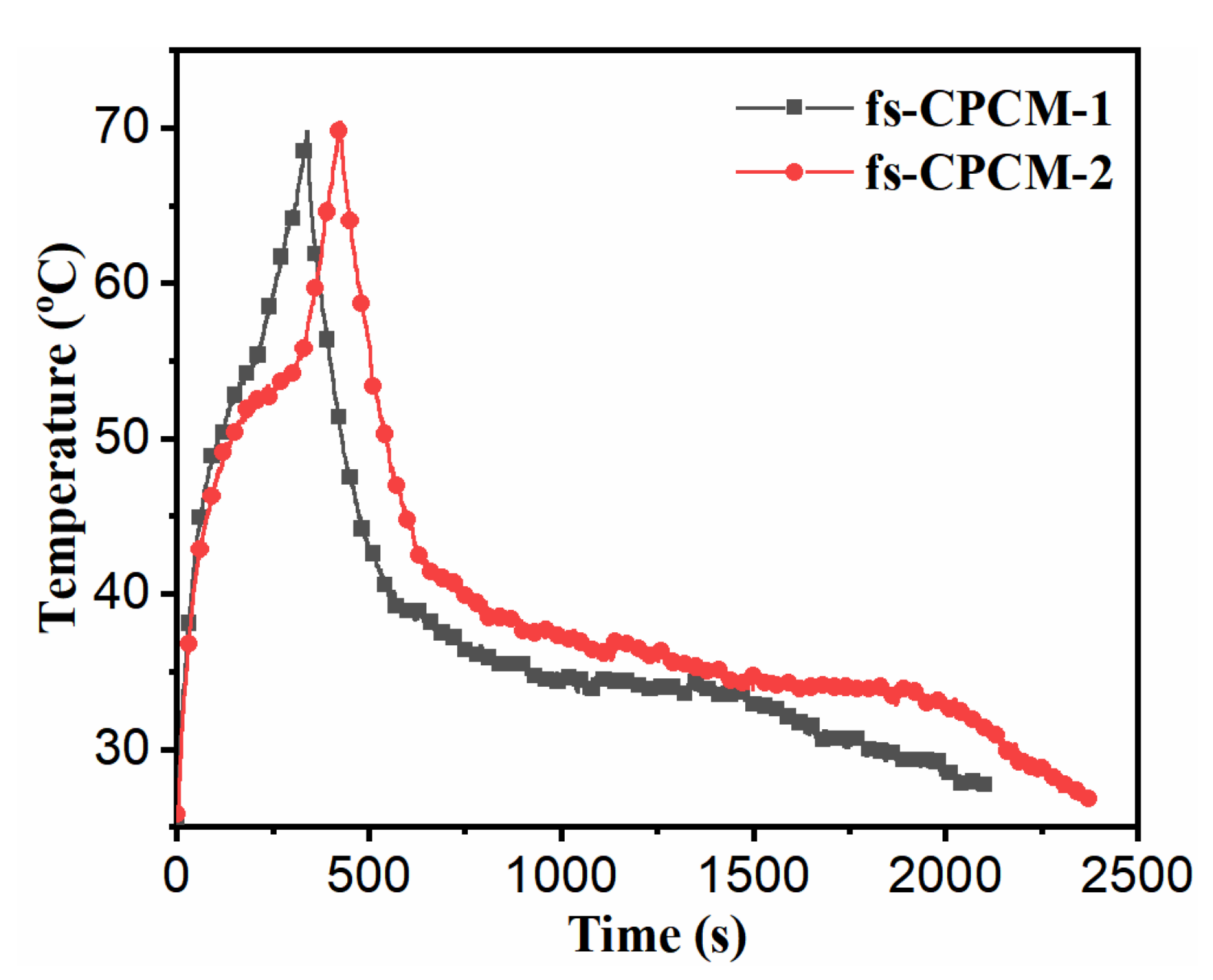
Temperature–time curves of the fs-CPCM-1 and fs-CPCM-2 during the heating and cooling process.

In order to study the light-to-thermal conversion behavior and solar thermal energy storage performance of fs-CPCM-1 and fs-CPCM-2, respectively, the simulated 500 W solar irradiation system and temperature acquisition system based on an infrared thermography camera were built by ourselves (Figure 9a), as described in our previous work [35]. Figure 9b,c and Figure 10 show the thermal images and temperature–time curves of the fs-CPCM-1 and fs-CPCM-2 during the heating and cooling processes, respectively. In Figure 9b,c, the blue and red parts represent low temperature and high temperature, respectively. Take fs-CPCM-1 as an example (as shown in Figure 9b), at the beginning (0 s), the entire image is closen to be blue in color, which indicates that the temperature of the sample approaches ambient temperature. With the opening of the irradiation source, all of the sample temperatures and ambient temperature rise rapidly, and the thermal image changes from blue to red (0 to 337 s). For fs-CPCM-1, in Figure 10, between 116 s and 215 s ($∆t_{1}$ = 99 s), there is a plateau due to the phase change, and the melting latent heat was stored. However, because of the lower phase change enthalpy for fs-CPCM-1 but the high irradiation intensity, the plateau is not very clear. At about 337 s, the fs-CPCM-1 sample temperature reaches 70 °C, the irradiation source switch was turned off. From 337 to 2100 s, all the sample temperatures and ambient temperature begin to drop rapidly, and the thermal image changes from red to blue. At about 570 s, the fs-CPCM-1 sample temperature drops to 40 °C, the melted PEG segment in fs-CPCM-1 begins to crystallize and release the stored latent heat, and, at the same time, the fs-CPCM-1 sample temperature was maintained between 35 and 40 °C (from 570 to 1400 s, $∆t_{2}$ = 830 s), which is higher than the ambient temperature. Therefore, in the thermal images at 480, 800 and 1300s, the color of the sample is redder than the ambient color (Figure 9a). The phase change of fs-CPCM-1 is completed at about 1400 s, and the stored latent heat is completely released. After that, the temperature of fs-CPCM-1 further drops at a fast rate and approaches ambient temperature (27 °C) at about 2100 s. 

For fs-CPCM-2, its light-to-thermal conversion behavior performance is similar to that of fs-CPCM-1. However, compared with fs-CPCM-1 ($∆t_{1}$ = 99 s), the plateau of fs-CPCM-2 is observed for a longer period during the melting process (from 116 to 317 s, $∆t_{1}^{\prime }$ = 201 s), which indicates that more melting latent heat was stored due to the high PEG segment loading in fs-CPCM-2. In addition, due to the high melting latent heat that was stored, the plateau of fs-CPCM-2 during the cooling process (from 740 to 1980 s, $∆t_{2}^{\prime }$ = 1240 s) also lasts longer than fs-CPCM-1 ($∆t_{2}$ = 830 s). The above results demonstrate that, compared with fs-CPCM-1, the fs-CPCM-2 has a larger phase transition latent heat, and superior performance in terms of light-to-thermal conversion, solar thermal energy storage, and waste heat recovery performance.

### 3.6. Waste Heat Recovery

**Figure 11:**
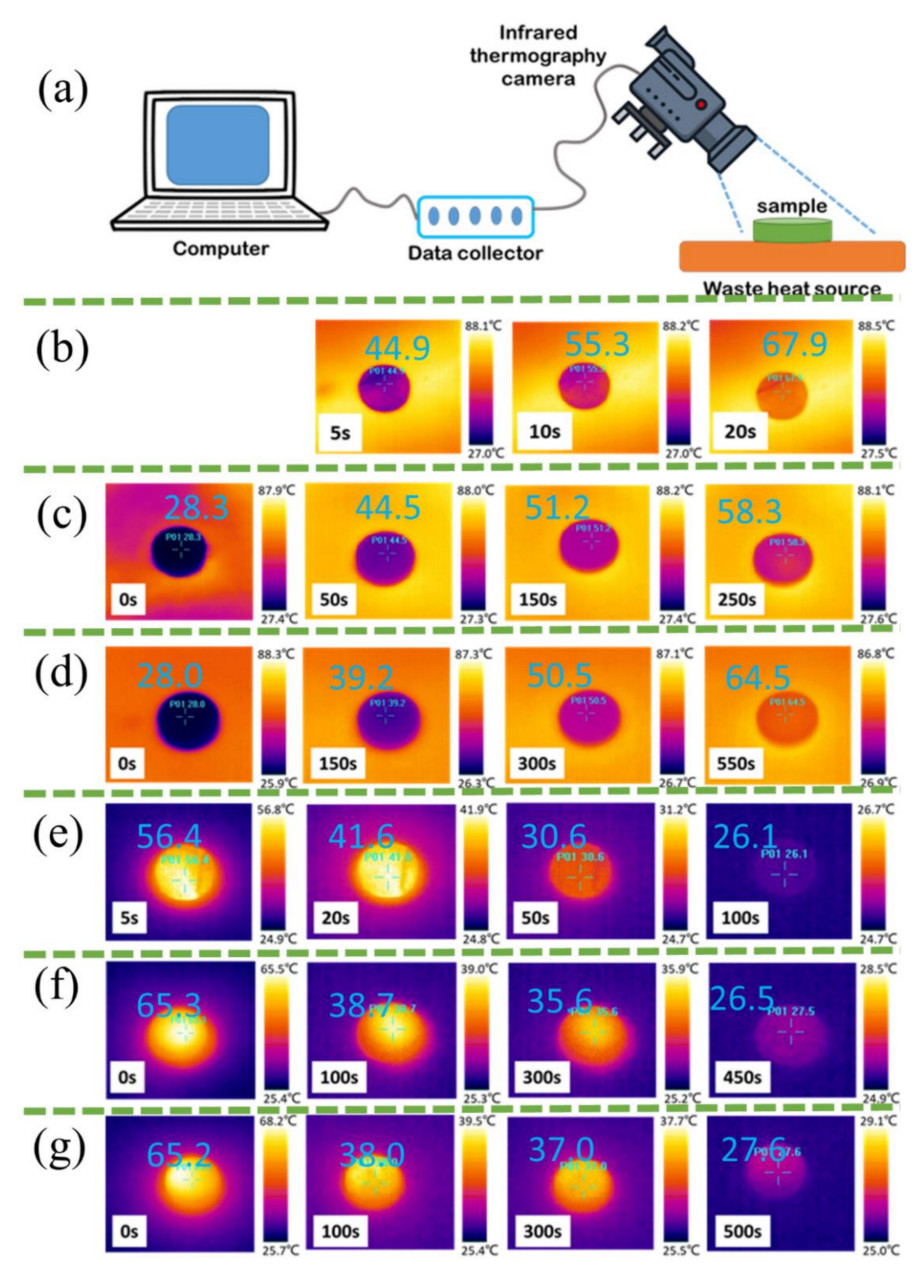
(**a**) waste heat recovery schematic, thermal images of heat recovery for (**b**) reference sample, (**c**) fs-CPCM-1, and (**d**) fs-CPCM-2, thermal images of heat release for (**e**) reference sample, (**f**) fs-CPCM-1, and (**g**) fs-CPCM-2 during the waste heat recovery and release process.

**Figure 12:**
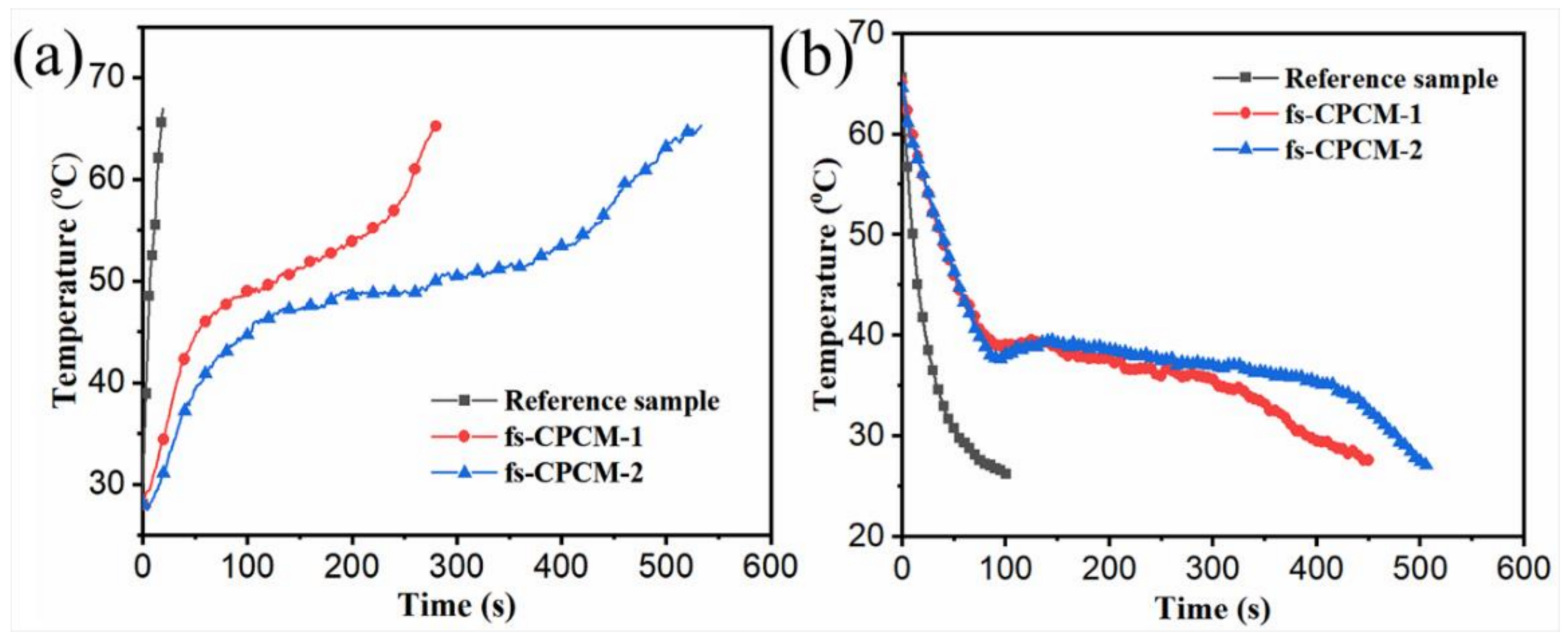
Temperature–time curves of the reference sample, fs-CPCM-1 and fs-CPCM-2 during the waste heat (**a**) recovery and (**b**) release process.

As is widely known, a large amount of low-grade thermal energy in nature is underutilized. If this energy can be stored and utilized by TES technology based on PCMs, the utilization efficiency of energy can be significantly improved. Hence, the homemade simulated waste heat recovery system and temperature acquisition system based on an infrared thermography camera were used to study the waste heat recovery behavior of fs-CPCM-1 and fs-CPCM-2 (as shown in Figure 11a). At the same time, in order to visually demonstrate the waste heat recovery function of the obtained fs-CPCMs, KBr that does not undergo a phase change between 20 and 100 °C was chosen as a reference sample. At the beginning, the samples were placed on a 90 °C heat source surface, respectively. Then, the sample will be heated by the heat source due to the temperature difference between them, and the sample temperature was recorded. When the temperature of the sample reaches approximately 65 °C, the sample is placed at a low ambient temperature and the variation of its temperature is followed through the infrared thermal imager. Figure 11b–g and Figure 12 show the thermal images and temperature–time curves of the reference sample, fs-CPCM-1, and fs-CPCM-2 during the heating and cooling process, respectively. Similar to Figure 9, in Figure 11b–g, the blue part stands for low temperature and the red part stands for high temperature. As shown in Figure 12a, the reference sample (KBr) temperature increases rapidly from ambient temperature (28.0 °C) to about 65.0 °C within 20 s. After removing it from the heat source, its temperature also declines rapidly from 65.0 °C to ambient temperature within 100 s. No plateau appears on its temperature–time curve during the heating and cooling process. It indicates that only the sensible heat and no latent heat is stored by the reference sample throughout this process. However, for fs-CPCM-1 and fs-CPCM-2, there are obvious plateaus in the temperature–time curves. Similar to the solar thermal energy storage and release process discussed above, the temperature plateaus in the heating and cooling processes correspond to the thermal energy storage and release processes, respectively. In addition, due to the higher phase transition enthalpy, the plateau length of the fs-CPCM-2 is longer than that of the fs-CPCM-1 in the temperature–time curves. This shows that the fs-CPCM-2 can store more thermal heat than fs-CPCM-1 in the same environment.

### 3.7. Thermal Stability

**Figure 13:**
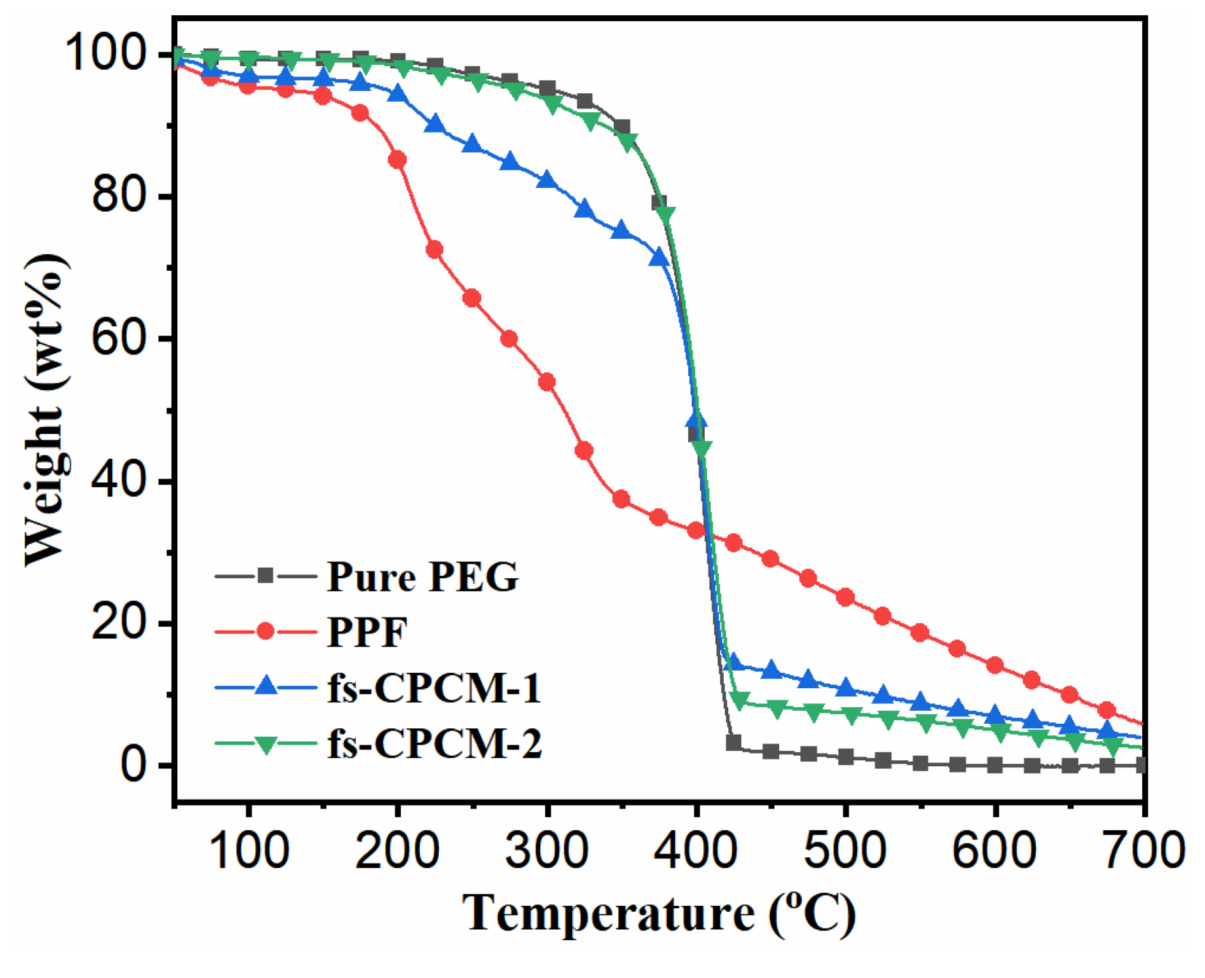
TGA curves of pure PEG, PPF, fs-CPCM-1 and fs-CPCM-2.

For fs-CPCMs in practical applications, the thermal stability is another important parameter. The TGA curves for pure PEG, PPF, fs-CPCM-1, and fs-CPCM-2 are displayed as Figure 13, and the corresponding thermal decomposition parameters were summarized in Table 4. As shown in Figure 13, there is only one thermal degradation stage for pure PEG at about 400 °C, but three thermal degradation stages were observed for PPF between 50–700 °C due to the complex composition. It is worth noting that the thermal stability of PPF is significantly lower than pure PEG. Therefore, as compared to pure PEG, the thermal stability of fs-CPCM-1 and fs-CPCM-2 is decreased by a greater or lesser extent with the introduction of PPF. As summarized in Table 4, the $T_{1}$ (the temperature at which 1 wt % degradation occurred), $T_{5}$ (the temperature at which 5 wt % degradation occurred), and $T_{max}$ (the maximum degradation temperature) of pure PEG are 199.5, 301.9, and 400.5 °C, respectively. However, the $T_{1}$ and $T_{5}$ of PPF are only 46.6 and 125.1 °C, and there are three $T_{max}$ (61.5, 208.8, 323.7 °C) for PPF, which corresponds to the three thermal degradation stages, respectively. Thus, with the introduction of PPF, the $T_{1}$ and $T_{5}$ of fs-CPCM-1 and fs-CPCM-2 are decreased to 60.1, 192.3, and 171.5, 280.4 °C, respectively. However, due to the low PPF loading, the $T_{1}$ and $T_{5}$ of fs-CPCM-2 are all higher than that of fs-CPCM-1. At the same time, compared to the pure PEG, there is no significant change in the $T_{max}$ for fs-CPCM-1 and fs-CPCM-2. Considering the long-term working temperature range of PEG-based fs-CPCMs (0–100 °C) and comprehensive performance, the fs-CPCM-2 does not undergo degradation during the phase change and has good thermal stability with a wide temperature range, thereby it can be directly used as a solar light absorber in solar thermal energy storage and waste heat recovery.

## 4. Conclusions

In this study, a series of novel bio-based PEG/PPF (fs-CPCM-1) and -NCO-terminated PEG/PPF (fs-CPCM-2) composites as stable phase change materials were successfully prepared through a simple vacuum adsorption by employing PPF as the matrix and PEG or -NCO-terminated PEG to induce a phase change. The leakage test reports that the maximum adsorbed mass fraction of PEG segment in the fs-CPCM-1 and fs-CPCM-2 were 49.2 and 89.9 wt %, respectively. The FTIR and SEM results identify that the excellent shape stability of fs-CPCM-1 during the phase change can be attributed to physical adsorption but should be attributed to both the chemical crosslinking structure and physical adsorption in the case of fs-CPCM-2. The XRD and POM results indicate that the crystalline regions of PEG segments in fs-CPCM-1 and fs-CPCM-2 were evidently restricted by the introduction of PPF, and the spherocrystal size was clearly smaller than that of pure PEG. However, their crystalline structures are not influenced by the introduction of PPF and a cross-linking network. The DSC, TGA, and accelerated thermal cycling test results indicate that, compared to fs-CPCM-1, the obtained fs-CPCM-2 has a higher latent heat and thermal reliability and stability over a long period of time. To summarize, the obtained novel bio-based fs-CPCM-2 demonstrates great potential for application in the fields of solar thermal energy storage and waste heat recovery.

## Figures and Tables

**Table 1:** The phase change parameters of pure PEG, fs-CPCM-1 and fs-CPCM-2.

Samples	$T_{mo}{(}^{{}^{\circ}}C)$	$T_{mp}{(}^{{}^{\circ}}C)$	$T_{me}{(}^{{}^{\circ}}C)$	$∆H_{m}(J/g)$	$T_{co}{(}^{{}^{\circ}}C)$	$T_{cp}{(}^{{}^{\circ}}C)$	$T_{ce}{(}^{{}^{\circ}}C)$	$∆H_{c}(J/g)$
Pure PEG	53.6	62.5	67.2	182.3	41.5	36.1	31.4	173.5
fs-CPCM-1	52.7	67.1	72.6	88.9	38.6	30.2	22.5	85.1
fs-CPCM-2	52.2	66.4	72.9	143.2	40.9	34.3	26.5	141.8

$T_{mo}$: onset melting temperature; $T_{mp}$: peak melting temperature; $T_{me}$: end melting temperature; $T_{co}$: onset crystallization temperature; $T_{cp}$: peak crystallization temperature; $T_{ce}$: end crystallization temperature.

**Table 2:** Phase change enthalpy of PEG-based fs-CPCMs in literature.

fs-CPCMs	$∆H_{m-PEG}\times W_{f}(J/g)$	$∆H_{m-PCM}(J/g)$	λ (%)	References
PEG/MDI/PVA	168.4	72.8	43.2	[29]
PEG/HDI/CO	167.6	117.7	70.2	[30]
PEG/MDI/Xylitol	162.8	76.4	46.9	[31]
PEG/HDIT	157.0	136.8	87.1	[22]
PEG/HDI/WP	136.4	134.2	98.4	[32]
PEG/CaCl/CNT	152.5	128.6	84.3	[33]
PEG/TTI	169.5	105.1	62.0	[34]
PEG/PPF	89.7	88.9	99.1	Present study
PEG/HDI/PPF	163.9	143.2	87.4	Present study

**Table 3:** The thermal reliability and reusability of fs-CPCM-1 and fs-CPCM-2.

Sample	$∆H_{mb}(J/g)$	$∆H_{ma}(J/g)$	$\eta_{m}(\%)$	$∆H_{cb}(J/g)$	$∆H_{ca}(J/g)$	$\eta_{c}(\%)$
fs-CPCM-1	88.9	87.6	1.5	85.1	84.7	0.5
fs-CPCM-2	143.2	142.8	0.3	141.8	139.9	1.3

**Table 4:** The thermal decomposition parameters of pure PEG, PPF, fs-CPCM-1, and fs-CPCM-2.

Samples	$T_{1}({}^{\circ}C)$	$T_{5}({}^{\circ}C)$	$T_{max1}{(}^{{}^{\circ}}C)$	$T_{max2}$	$T_{max3}$
Pure PEG	199.5	301.9	400.5	−	−
PPF	46.6	125.1	61.5	208.8	323.7
fs-CPCM-1	60.1	192.3	400.1	−	−
fs-CPCM-2	171.5	280.4	400.4	−	−
